# Compartment-specific regulation of stromal differentiation of the spleen in health and disease

**DOI:** 10.1038/s41435-026-00411-4

**Published:** 2026-08-25

**Authors:** Chayeen Brotzki da Costa, Angéla Kecskés, Tibor Jánosi, Kata Váradi, Zoltán Kellermayer, Péter Balogh

**Affiliations:** 1 Department of Immunology and Biotechnology, University of Pécs Medical School, Pécs, Hungary; 2 Department of Pharmacology and Pharmacotherapy, University of Pécs Medical School, Pécs, Hungary; 3 Nano-Bio-Imaging Core Facility, University of Pécs Medical School, Pécs, Hungary; 4 Szentágothai Research Centre, Multiple myeloma microenvironment research group, Pécs, Hungary

**Keywords:** Spleen, Cellular immunity

## Abstract

Peripheral lymphoid organs in mammals are highly organized, complex tissues that support adaptive immune responses. In terms of comparative developmental biology, the mobile leukocytes of shared hematopoietic lineages in the spleen, lymph nodes, and mucosal lymphoid tissues perform similar functions during antigen recognition and intercellular communication, indicating them as largely homologous. However, the sessile stromal elements within these tissues that create preferential lodging domains for leukocytes differ considerably in their developmental features and structural organization, appearing analogous rather than homologous. Recent studies of splenic stromal architecture have identified region-specific transcription factors, chemokine signatures, and cell surface molecules that guide the specialization of the red pulp and marginal zone, two compartments unique to the spleen. This review—with particular emphasis on Nkx family members as key morphogens regulating spleen development and function—summarizes the roles of critical transcription factors in stromal differentiation pathways, architectural features, and immunological processes under both physiological and pathological conditions.

**Figure:**
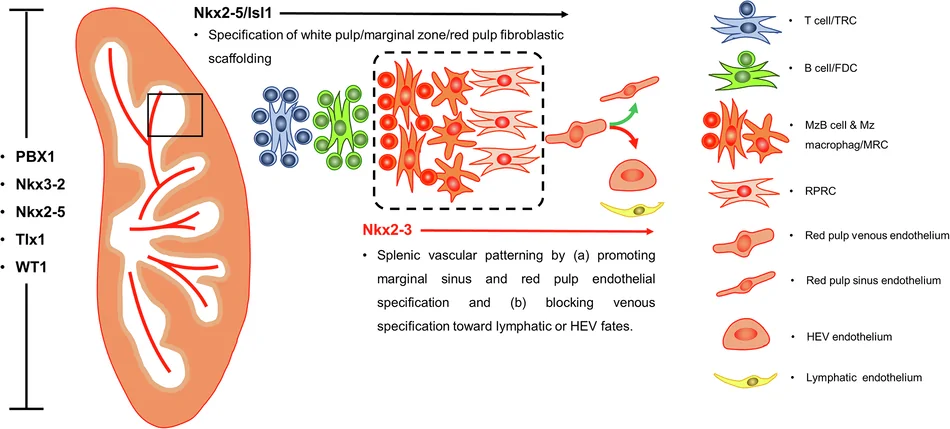

## Introduction: overview of spleen structure, developmental features and organ-specific functions

Although traditionally categorized in the same group of peripheral or secondary lymphoid organs (SLO) engaged in hosting immune responses, the spleen, lymph nodes, and mucosa-associated lymphoid tissues differ considerably in several aspects. As a solitary organ for blood filtering, the spleen is present in all vertebrates, whereas lymph nodes appear typically in mammals, draining tissues in both external and internal surfaces via lymphatics; on the other hand, mucosa-associated lymphoid tissues in birds and mammals receive antigens through transepithelial uptake without any circulatory transport. In addition, these organs also differ in number and tissue architecture, comprising compartmentalized leukocyte subsets. Of these sites, the spleen is the largest single peripheral lymphoid tissue with complex immunological functions, including local and systemic immune reactions, B-cell specification towards follicular and marginal zone B-cell subsets, and homeostasis, besides extramedullary hematopoiesis. These different features can be linked to distinct tissue regions of the spleen [1].

Although the overwhelming majority of spleen cells are comprised of various mobile and sessile hematopoietic subsets, their arrangement into separate compartments is organized by non-hematopoietic mesenchymal cells, collectively referred to as stromal cells. Beyond providing structural scaffolds, stromal cells play significant roles in lymphocyte homing and survival, maintenance of immunological responsiveness, and tissue restoration following germinal center (GC) formation [2]. Furthermore, during its ontogeny, the spleen functions as an auxiliary hematopoietic organ during the embryonic period and postnatally in stress hematopoiesis; therefore, its stromal complexity also includes sites functioning as hematopoietic niches, absent in other peripheral lymphoid tissues [3]. In the following sections, we outline the main structural components of the spleen, its stromal constituents underpinning various lymphoid domains, and their compartment-specific developmental characteristics, including their transcriptional determinants that guide the emergence of the spleen and its regions. In the last part, we summarize the effect of aging on the stromal integrity and possible reversal for regaining immunological competence.

### STROMAL landscape of the spleen: compartment-specific complexity mirroring hematopoietic segregation

The basic structural components of the spleen include the red pulp and white pulp, where the two domains are separated by and connected through the marginal zone (MZ). This rim in mice comprises specialized MZ B cells and MZ macrophages, arranged around marginal reticular cells (MRCs) and a MAdCAM-1^+^ marginal sinus, absent in humans [1].

In young adult mice, the bulk of white pulp stromal components can be identified based on their Bst-1 (CD157/BP-3) display, whereas Ly6C1^+^/desmin positive stromal cells colonize the red pulp [4, 5]. Within the white pulp, T cells accumulate around the arteriolar branches of the splenic artery, forming the periarteriolar lymphoid sheath (PALS), also containing dendritic cells (DCs) and fibroblastic reticular cells (FRCs), producing CCL21 as a T-zone homeostatic chemoattractant [6] and IL-7 for supporting naive T-cell survival [7]. Similar to lymph nodes [8], splenic FRCs also form extracellular matrix (ECM)-based tubular sieves called conduits, which facilitate CCL21 T-cell tropic chemokine dispersion and gradient formation as directional cues [9] for T cells and dendritic cells. A special white pulp region where the PALS directly abuts the MZ without intervening B-cell follicles is the bridging channel, which facilitates T-cell migration from the red pulp to the PALS along local stromal cells [10]. Here, the local CD4^+^ cDC2 subset of dendritic cells—positioned by EBI2 [11] and retained via CD97-CD55 recognition—can interact with CD4^+^ T cells and select for follicular T helper (T_FH_) commitment, thus supporting B-cell activation and humoral immune responses [12].

The B-cell follicles along the PALS are arranged around a scaffold formed by non-migratory follicular dendritic cells (FDCs) of mesenchymal origin, secreting the B-cell chemoattractant CXCL13 [13]. Furthermore, they display a range of surface receptors for long-term antigen retention, including Fc receptors CD16/32 and CD23, as well as complement receptors CD21 and CD35. Although sessile, FDCs can alter their VCAM-1/MAdCAM-1 expression during germinal center reaction [14], parallel to the emergence of other stromal subsets, as defined by their follicular positioning relative to the T-zone, blunted expression of FDC-associated markers, and increased CXCL12 chemokine production [15].

The MZ stromal cells include the MRCs co-expressing RANKL/Tnfsf11 and MAdCAM-1. In the late prenatal/early postnatal period, this area also serves as a source for perivascular reticular cells (PRC) as a common precursor for MRCs and FDCs emerging in a stepwise manner [16, 17]. MRCs also communicate with splenic Type 3 innate lymphoid cells (ILC3s) expressing RORγT via BAFF, CD40L, and Notch-ligand Delta-like 1 (DLL1) to enhance T-independent immune responses by MZ B cells [18]. MZ macrophages are recruited and retained as sessile cells within the MZ in a CCL19/CCL21-dependent fashion, and are important in the organization of MZ architecture [19]. The CD169-positive metallophilic macrophages adjacent to the white pulp form the inner ring of the MZ with a more pronounced density over the follicles, whereas the outer ring contains the MZ macrophages displaying the Macrophage Receptor with Collagenous structure (MARCO) scavenger receptor and Specific Intracellular Adhesion Molecule-Grabbing Nonintegrin R1 receptor (SIGN-R1) [20]. MZ macrophages also communicate with the white pulp via conduits for the follicular distribution of FDC-derived CXCL13 and MARCO from MZ macrophages [9, 21]. Additionally, MZ B cells rely on a CD55-CD97 interaction-dependent orientation mechanism, which defines their location and responsiveness to blood-borne pathogens [22] while actively shuffling between the MZ and follicles [23]. Furthermore, SIGN-R1^+^ MZ macrophages support germinal center reactions by guiding dendritic cell assistance for T_FH_ cells [24]. Importantly, the MZ also acts as an egress site for follicular B cells involving S1PR1, draining towards the red pulp [23], whereas S1PR3 influences the organization of MAdCAM-1-positive marginal sinus and MZ macrophage architecture [25].

The red pulp (RP) is dominantly an erythromyeloid compartment of the spleen; however, it also serves as an important domain for hosting plasma cells and transitional B cells as recent emigrants from the bone marrow, for completing their differentiation and selection into follicular vs. MZ B cells [26, 27]. Although with less clear structural organization compared to white pulp or MZ, here the majority of sessile cells include RP macrophages and various RP-associated stromal cells. Ly6C1⁺/desmin⁺ stromal cells, as a mesenchymal framework, express the spleen morphogenic factor WT1 and surround MECA-32⁺/PDGFRα⁻/WT1⁻ endothelial cells. These WT1-dependent stromal cells uniquely support local macrophage clustering through CCL2/CCL7- and Csf1-dependent mechanisms [28], alongside the PPARγ/SpiC dependence of red pulp macrophages [29]. Those clusters are capable of supporting extramedullary hematopoiesis from local precursors and participate in the removal of effete red blood cells [30]. This latter function is also closely related to the splenic Fe-recycling in organismal iron homeostasis [31]. The auxiliary hematopoietic functions thus render the splenic RP similar to the bone marrow, probably reflecting the prenatal spleen’s role as auxiliary primary hematopoietic tissue [32, 33].

**Fig. 1:**
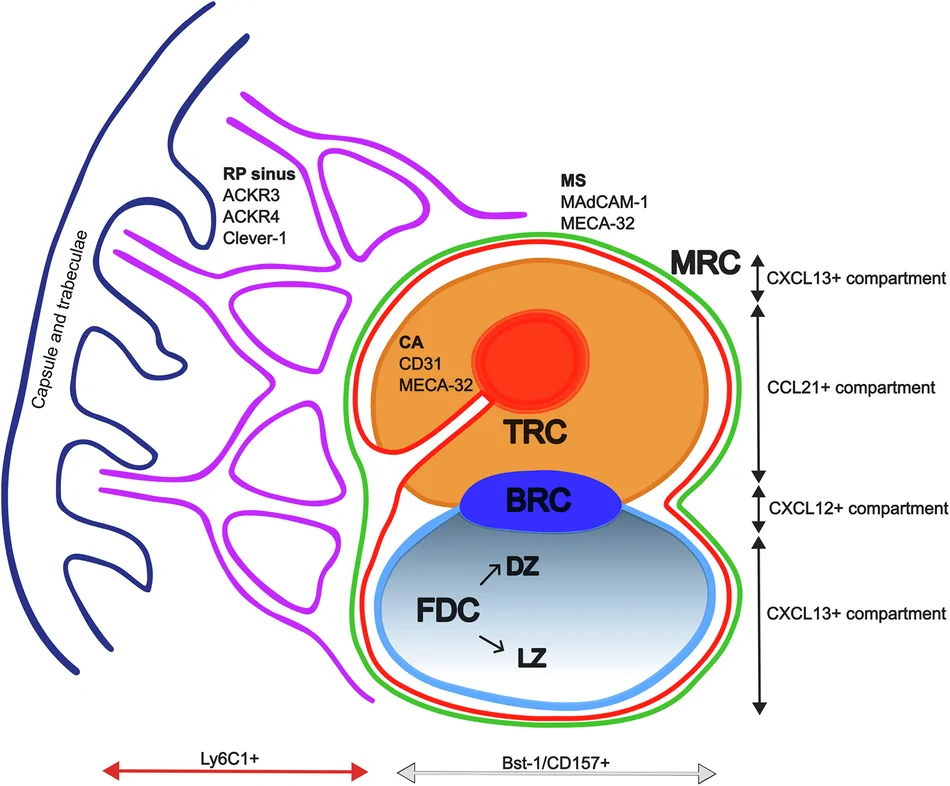
Compartmentalization of mouse splenic stromal subsets. From left to right, the dark blue lines label the capsule and trabeculae, latter adjacent to the red pulp (RP) sinus meshwork, characterized by the markers ACKR3, ACKR4, and Clever-1 and depicted in purple. Neighboring this, the marginal sinus (MS) is represented by the markers MAdCAM-1 and MECA-32. The green line defines the marginal reticular cells (MRC) zone, a compartment defined by CXCL13 positivity and located next to the red line that represents the terminal segment of arterial supply from the central arteriole (CA), which branches into the follicle and has CD31 and MECA-32 as markers. The T-cell zone reticular cell compartment (orange-colored region) is CCL21-positive and is situated close to the B-cell zone reticular cells (BRC), depicted as a dark blue region and identified as a CXCL12+ compartment. It is located closely to the follicular dendritic cell (FDC) zone, designated as a CXCL13+ compartment, and upon germinal center (GC) formation, separates into dark and light zones (DZ and LZ, respectively). At the bottom, the red arrow denotes the region of the scheme representing the red pulp, associated with Ly6C1 + ; the remaining regions represent the white pulp, indicated as Bst-1/CD157 + .

In addition to the diverse hematopoietic and fibroblastic reticular components, the white pulp, MZ, and RP have a distinctive vasculature, containing different types of blood vessels, including arterioles, sinuses, and venules, lined by subset-specific endothelium with distinguishable phenotypic and functional traits [34–37]. While the white pulp is relatively devoid of blood vasculature, the RP is richly perfused; here, atypical chemokine receptors ACKR4 and CXCR7/ACKR3 may modify local CCL19 and CXCL12 gradients, respectively, thus influencing the splenic turnover of lymphocytes [34, 38]. Overall, splenic compartmentalization involves segregation of T and B cells, alongside specialization of phenotypically and functionally distinct sessile reticular and vascular networks across splenic domains to support lymphocyte recirculation, chemotactic positioning, survival, and humoral immunity. Figure 1 summarizes this organization by illustrating the major fibroblastic reticular cell subsets and principal vascular segments of the mouse spleen.

### Region-specific emergence of stromal architecture of the spleen: subsets defined by scRNAseq, phenotypic traits, and developmental trajectories

The previous section summarized the cellular organization of various compartments of the splenic mesenchyme and their main roles in organizing the lymphoid architecture in the adult mouse spleen as a reference. Although several phenotypic markers proved very useful in dissecting their spatial arrangements, the complex spectrum of differentiated splenic stromal subtypes, as well as their developmental relationships, has remained undefined until recently. By improving tissue extraction protocols and using multiparametric lineage-tracing approaches for stromal subpopulations in chemokine Ccl19-reporter mice, scRNA analyses of murine splenic stromal cells have revealed a previously unknown complexity emerging in the postnatal period. In this part, we review the assignment of stromal subsets corresponding to their microanatomical locations and functions.

Earlier analyses have established the commitment and subsequent specialization of perivascular mesenchymal reticular cells (PRC) from mesenchymal lymphoid tissue organizer (mLTo) progenitors expressing Nkx2-5/Islet1 transcription factors [39] into T-zone FRC, MRC, and FDC subsets shortly before birth and during the first postnatal week, requiring lymphotoxin beta-receptor (LTβR) activity [17]. Lineage-tracing and scRNA-seq analyses in Ccl19 reporter mice revealed that Ccl19-traced PDGFRα/Sca-1 (Ly6A1)/CD105-positive progenitors give rise to the white pulp mesenchymal stroma, including the follicular, T-zone, and marginal zone (MZ) compartments [4]. The late embryonic commitment and gradual maturation of murine mLTo cells begin shortly before birth and continue throughout the first postnatal week, following a gradient along the arterial tree, with a progressively reduced degree of maturation toward smaller branches [4]. This pattern of peri- and early postnatal splenic architecture in mice (up to postnatal week 1) may correspond to the developmental state of the human fetal spleen at approximately 18–20 weeks of gestation [40].

The subsequent differentiation of mesenchyme into stromal subsets is associated with clonal proliferation of precursors within local microenvironments during interactions with hematopoietic cells, together with the gradual switch of mRNA expression from organogenesis-associated genes to specified markers characteristic of PRC, MRC, FRC, and FDC subsets in an LTβR-dependent manner. Based on global scRNA-seq analysis, this differentiation results in eleven distinct cell clusters with six white pulp clusters harboring Ccl19-marked mRNA expression, including two novel subsets of CD157⁻/Pdpn⁻/Sca-1⁻ triple-negative cells (TNCs) maintained independently of LTβR signaling [4]. These studies confirmed the earlier findings on the perivascular/adventitial cells as an important stromal niche for the genesis of splenic mesenchymal scaffolding [16, 17].

Subsequent studies have further expanded the spectrum of diverse subpopulations of non-endothelial stromal cells in the adult murine spleen, with distinctive microanatomical, phenotypic, and functional characteristics [5, 41], with largely similar subset identification and putative classification, despite slightly different experimental approaches employed. Subdivision of the previously defined FRC, MRC, and FDC populations identified more than ten stromal subsets within the white pulp. Of these, five are follicular stromal subsets (FDC and BRCs), two are marginal zone (MRC and bridging channel reticular cells [BCRC] subsets, and three are T-zone reticular cells (TRC), with an additional subset with shared T-B-zone features (TBRC). Furthermore, the splenic adventitial reticular cells (ARCs) and mural cells also demonstrated a highly complex composition that could be resolved into five distinct subsets [41]. Of these, two clusters of ARCs expressing *Clec3b*, CD34, and Sca-1/Ly6C1 may serve as undifferentiated precursors for the fibroblasts in the white pulp and non-endothelial stromal cells of red pulp, closely associated with further two pericyte subsets expressing PDGFRβ, and a third mural cell subset distinguishable by the glial marker S100a. The maturation of several white pulp fibroblast subsets requires the SpiB transcription factor. Importantly, the human spleen also demonstrated a similar stromal complexity of fibroblastic scaffolding, although with different marker profiles, including the lack of Pdpn by FRCs, increased expression of CD157 by CD105-positive red fibroblasts, and a relatively lesser expression of FDC-associated CD21 [41].

In addition to the extensive complexity of white pulp macrophages, the red pulp also harbors a diverse set of reticular fibroblasts [4, 5, 41]. The four subsets of Ly6C1^+^ RP stromal cells with various heme oxygenase-1 (HMOX1) production comprise the bulk of the RP stromal meshwork, also extending into the MZ. Its mutation, denoted as OMIM #614034, is associated with asplenia [42]. Furthermore, a distinct group expressing Melanoma Cell Adhesion Molecule (Mcam/CD146) was assigned to the perivascular area, divided into further subsets according to their CXCL13 and vascular smooth muscle characteristics [5], probably corresponding to the pericytes/mural cells [41]. In addition, an adventitial CD34^+^ cell subset around the Mcam^+^ subset could also be defined as a fourth cluster [5], similar to ARCs [41]. The constituents of RP reticular cells are likely to represent diverse differentiation stages and/or activation states, rather than lineage affiliation, as evidenced through their interferon-stimulated gene (ISG) expression [28, 41].

Although sessile stromal cells in lymphoid organs have long been considered primarily structural and developmental components, recent evidence has revealed their surprising plasticity in adapting to immune responses. Following lymphocytic choriomeningitis virus Armstrong strain (LCMV Arm) acute infection, a significant divergence of gene expression by T-zone reticular cells (TRCs) and MRCs was noted at day 8 after infection, but PRCs showed significantly fewer differentially expressed genes. Genes highly up-regulated in TRC and MRC after infection included cell cycle and antigen processing and presentation pathway genes, as well as several chemokines and cytokines. On the other hand, down-regulated genes after infection were enriched for ECM and angiogenesis, neuron differentiation, in addition to homeostatic chemokines *Ccl19* and *Ccl21a* and cytokines *Il7* and *BAFF* [41]. Furthermore, following persistent infection with LCMV clone 13 (CL13), which differs by two amino acids from LCMV Arm, RNA-seq revealed that substantially fewer genes were differentially expressed by TRC and MRC after CL13 infection. These findings demonstrate that acute viral infection induces dynamic transcriptional reprogramming in splenic FRC subsets, thereby promoting host responses distinct from those associated with persistent viral infection [41]. Interestingly, infection with LCMV WE strain resulted in the destruction of splenic FRC architecture, associated with the reduction of *Ccl19*, *Ccl21a*, and *Il7* mRNA and an increase in the mRNA of the tumor necrosis factor (TNF) receptor/ligand family members, including LTα, LTβ, LTβR, LIGHT, TNFRI, and TNFRII, together with *Vcam1*, *Icam1*, and β_2_ integrin mRNA. Subsequent restoration of lymphoid architecture and the capacity to support immune responses required the presence of LTi cells and their LTβR-dependent interaction with stromal precursors [43].

Importantly, immune response-related alterations of stromal cells appear to overwrite tissue-specific traits, as evidenced by BRC scRNA signature differences and similarities between resting and immunized conditions using Cxcl13-reporter mice [44]. While stromal subset-specific traits were noted even within one (i.e., splenic or lymph node) organ-derived stromal subpopulations, including FDCs, MRCs, TBRCs, and pan-fibroblast marker Pi16 + [45] adventitial reticular (ARC) subset, the local naive B cells, GC B cells, T_FH_ cells, plasma cells, macrophages, and DCs showed no lymphoid tissue origin-related transcriptome segregation [46]. However, upon activation, B-zone reticular cells demonstrated an extensive spectrum of subset-specific, but lymphoid organ-independent alterations of mRNA signature, including NF-κB signaling, cytokine and chemokine production affecting leukocyte distribution, differentiation, and angiogenesis [44]. Collectively, these studies have unequivocally established the existence of site-specific FRC heterogeneity with largely corresponding subsets; however, future understanding could undoubtedly benefit from a conclusive nomenclature for stromal constituents, including their origin, eventual subset variants, functional specialization, and resting/active status [47].

### Development of the spleen: convergence driven by transcriptional regulation and stepwise fine-tuning by homeostatic chemokines and TNF family members

The preceding sections described the constituents of the highly complex stromal architecture of the spleen. In this section, we summarize the transcriptional programs that govern the development of the spleen as a distinct organ.

The initial specification of the spleen anlage is closely related to the emergence of the dorsal pancreas, regulated by the TALE-class homeodomain transcription factor Pbx1 [48] (reviewed recently in [49]), initiated by the appearance of a bilateral mesodermal cell layer as a transient organizer. This region is denoted as splanchnic mesodermal plate (SMP), and surrounds the midline-positioned stomach, whose appearance is blocked by the Dominant hemimelia (Dh) mutation in mice [50]. Next, Bapx1/Nkx3-2 promotes the mesenchymal condensation and leftward growth of the splenic anlage in the dorsal mesogastrium, thus separating it from the dorsal pancreas during the embryonic period between e9.5 and e12.5 days in mice [51, 52].

The spleen-specific mesenchymal differentiation within the dorsal mesogastrium segment of the splanchnic mesoderm is initiated by a regulatory loop connecting the transcription factors Hox11/Tlx1 and WT1, under the influence of Pbx1, as well as through an autoregulatory activation of Hox11 itself. Pbx1 also appears to influence the expression of Nkx2-5 [48], a major cardiogenic transcription factor. An essential element of the regulatory functions of Nkx2-5 is linked to the inhibition of p15INK4b, a member of the INK4 family of cyclin-dependent kinase inhibitors [53]. Importantly, Nkx2-5 and Islet1 (Isl1)-positive early splenic mesenchymal cells are closely linked to the development of the pancreas within the spleno-pancreatic mesoderm, serving as precursors for virtually all splenic stromal cells, except for the vascular endothelium [39]. As mentioned earlier, the differentiation of mesenchymal precursors into white pulp stromal cells also requires the SpiB transcription factor, as its deletion by a Ccl19-driven Cre/Lox approach leads to a substantial loss of T-zone FRCs and MRC, but without affecting FDCs [41].

In addition to the Nkx2-5-related path of splenic differentiation, another spleen-initiating trajectory is promoted by Nkx3-2/Bapx1 independently of Pbx1, but also connecting to the Hox11/WT1 pathway, indicating that the initiation of spleen organogenesis proceeds along several parallel pathways. Interestingly, both the single and combined heterozygosity of Pbx1 and Hox11/Tlx1 displayed frequent macroscopic abnormalities of the spleen while preserving tissue architecture, suggesting the importance of gene dosage in determining the normal formation of the spleen [48]. In a spatiotemporally restricted transcriptional loop, TLX1—influenced by nuclear receptor steroidogenic factor-1 (SF-1)—controls retinoic acid (RA) metabolism in the deeper parenchymal region of the splenic anlage by regulating *Cyp26b1* expression through binding to its AP-1 motif. Cyp26b1 enzyme is involved in the degradation of RA; its absence results in severe caudal malformations of limbs, intestines, and embryonic or postnatal lethality [54]. Thus, the lack of TLX-1 or its upstream regulator SF-1 leads to elevated RA signaling due to reduced degradation, which may ultimately inhibit splenic mesenchymal cell proliferation and VEGF-A–dependent vasculogenesis. In addition, dysregulated RA production may also affect the expression of *Rara* and *Rxra* nuclear receptors [55, 56].

The second stage of the prenatal spleen development is the emergence of local hematopoiesis from the sequential waves of yolk sac- or fetal liver-derived precursors, coupled with the gradual formation of white pulp and red pulp territories. The appearance of the white pulp is initially independent of the activity of LTβR [57], even though the subsequent separation of follicles and appearance of MZ are critically dependent on LTβR activity [58]. Interestingly, the differentiation of the CD169^+^ metallophilic macrophages forming the inner layer of MZ macrophages also requires ligand binding by RANK, where MZ stromal cells provide RANKL [59]. On the other hand, constitutive activation of alternative NF-κB signaling induces the dysregulation of MZ structure and the appearance of ectopic peripheral lymph node addressin (PNAd)-positive high endothelial venules (HEVs) in the red pulp [60].

The emergence of compartmentalized WP and MZ architecture, as well as the appearance of FDCs and the capacity to launch T-dependent immune responses, represents the third phase of white pulp fine-tuning. This can be further divided into four distinct stages, guided by CCL19 and CXCL13 chemokines, and stepwise acquisition of LTαβ_2_-dependence for their induction [17] in about 2-3-day intervals in postnatal mice [61]. In this process, first CXCR5/CCR7^+^ B cells and also lymphoid tissue inducer (LTi) cells colonize the splenic anlage, which is necessary to subsequently trigger LTβR-dependent induction of CCL19 production and expansion of desmin^+^/αSMA^+^ perivascular stromal cell precursors, although in the spleen, LTi cells appear redundant [62]. The third event is the increase of CXCL13 production, leading to the functional and physical segregation of CCL19/21^+^ T-zone-primed stromal precursors, as well as the CXCL13-producing FDC precursors from perivascular precursors [16], together with the congregation of B cells from their previous ring-like arrangement, and the enlargement of the T-cell zone. The continued LTβR engagement is necessary for promoting further maturation of reticular scaffolding, as evidenced by the acquisition of subset-specific stromal markers, Pdpn for T-zone FRC, and CR1/2 for B-zone FRC/FDC, respectively [4, 17].

After spleen morphogenesis is completed, the potential to initiate germinal center responses requires additional stromal–leukocyte interactions. The recognition of soluble tumor necrosis factor (TNF) from B cells by p55/TNFR1 expressed by splenic stromal cells is needed for the proper positioning of immature FDCs and marginal sinus-lining MAdCAM-1-positive cells [63–66], as well as CD30 and Pdpn for T/B segregation [67]. In addition, sphingosine 1-phosphate receptor 3 (S1P3) is necessary for the proper alignment of MAdCAM-1-positive endothelial cells, metallophilic macrophages, and MZ B cells [26].

### Nkx2-3 AND Nkx2-5—a differently similar relationship in spleen organogenesis

As discussed above, the transcriptional regulation of spleen mesodermal differentiation involves several Nkx family genes. Of these, the vertebrate *Nkx2-3* and *Nkx2-5* genes (*NKX2-3* and *NKX2-5* in humans) are two syntenic NK-2 class homologues of the Drosophila *tinman* cardiogenic homeodomain (HD) transcription factor, with similar structural and biochemical characteristics, but with diverse developmental roles [68]. Generally, these factors may have partly shared putative target DNA sequences recognized by the helix-turn-helix tertiary structure of their HD domain; however, their associating molecules determine their different effects in terms of both sequence specificity and upregulation versus inhibition of the target genes [69]. In this part, we compare the impact and developmental roles of these two transcription factors in the stromal specification of the spleen.

**Fig. 2:**
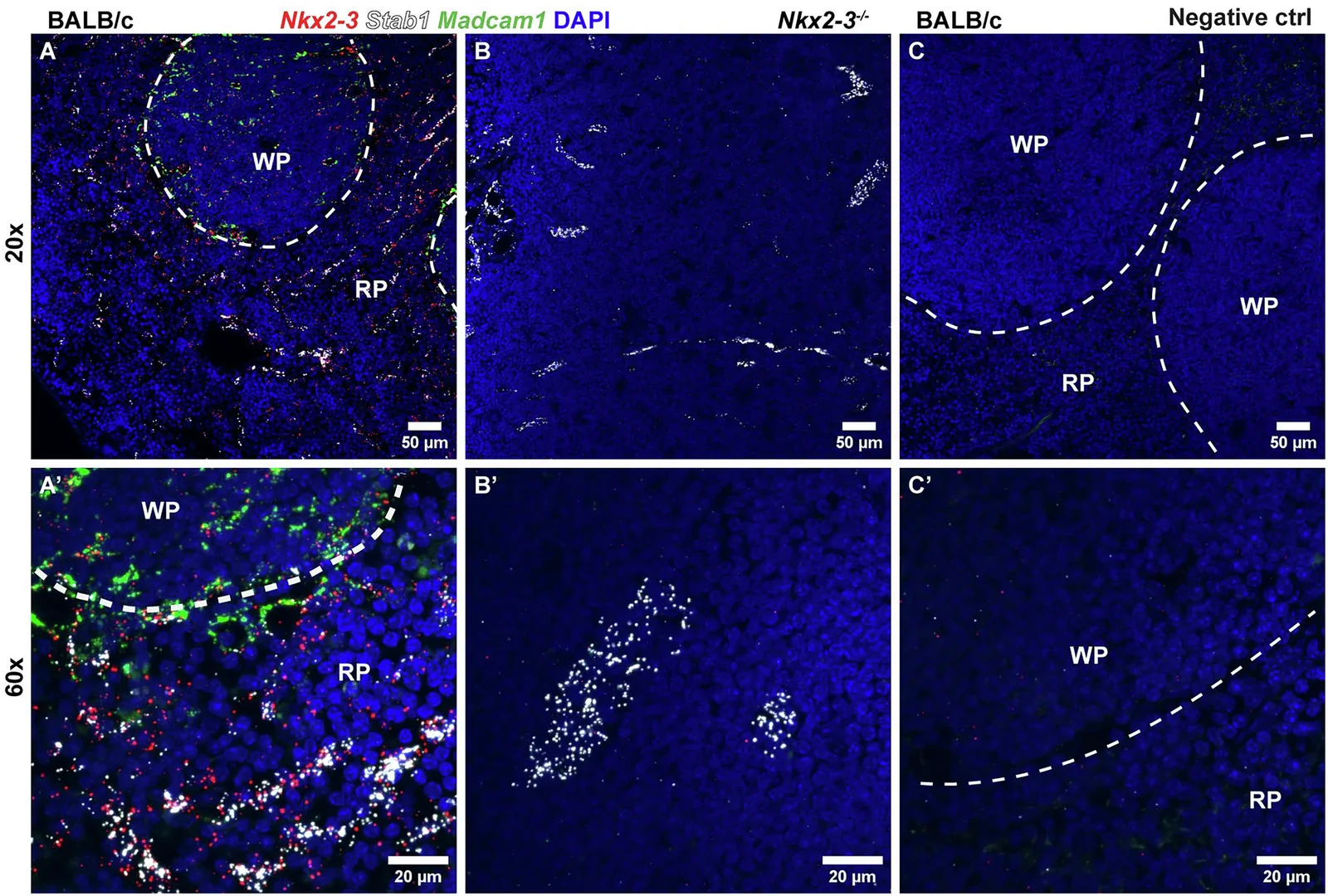
Regional expression preference of *Nkx2-3* mRNA in mouse spleen. **A** *Nkx2-3* mRNA (red) is partially co-localized with the red pulp–specific *Stab1* (white) and the marginal zone–specific *Madcam1* (green) mRNA in the spleen of naïve BALB/c mice, imaged with a 20x objective counterstained with DAPI. **A′** Higher-magnification image of the same region acquired with a 60x objective. **B** *Nkx2-3* mRNA expression is absent in the spleen of *Nkx2-3*^*–/–*^ mice; similarly, no *Madcam1* signal is detected, imaged with a 20x objective. **B′** Higher-magnification image of the same region acquired with a 60x objective. **C** As a negative technical control, RNAscope probe diluent was used in the spleen of naïve BALB/c mice, imaged with 20x or **C**’ 60x objective. Scale bars: 50 µm (**A**, **B**, **C**) and 20 µm (**A’**, **B’**, **C’**). Abbreviations: white pulp (WB), red pulp (RP). Brightness and contrast adjustment and *z*-projection of 60x images (10 stacks/image, 1 µm-intervals) were processed using maximum-intensity projection of the individual channels in Fiji.

In contrast to some forms of *Nkx2-5* mutations, the absence of *Nkx2-3* does not result in asplenia but causes a profound deviation from normal splenic structure. Nkx2-3–deficient mice exhibit smaller spleens with an ill-defined red pulp–white pulp boundary, accompanied by reductions in MZ macrophages, MZ B cells, a diminished MAdCAM-1–positive marginal sinus, and a drastic loss of red pulp macrophages [70, 71]. While earlier observations analyzing the spleen could not detect *Nkx2-3* mRNA expression in mature hematopoietic cells, recent data indicate that it is expressed in embryonic [72] as well as adult long-term reconstituting hematopoietic stem cells (LT-HSC), where it influences the maintenance of the LT-HSC subset by regulating mitophagy [73]. On the other hand, the structural defects and impaired B-cell responsiveness in *Nkx2-3*-deficient mice are linked to non-hematopoietic cells [74] and are reminiscent of the absence of LTβR deficiency; however, in the latter case, the RP appears unaffected, whereas in Nkx2-3-deficient mice, the RP vascular organization is missing [75]. Interestingly, expression of *Nkx2-3* mRNA is also a prominent marker distinguishing spleen vs lymph node T-zone fibroblasts and MRC subsets [41]. In our study, we were unable to identify a reliable reagent for immunohistochemistry to detect murine Nkx2-3 protein, despite validation attempts using Nkx2-3–deficient mice as a negative control, although in human spleen, we observed labeling of RP sinus endothelial cells for NKX2-3 displaying CD31 and von Willebrand factor (vWF) [76]. This expression pattern matches the RP vascular domain affected by Nkx2-3 deficiency in mice. Here, it is also important to remember that in this affected area in both humans and mice, MAdCAM-1 expression is typically absent, but contains a Stab1-encoded hyaluronan receptor Clever-1-dependent vascular exit bed for B cells and CD4^+^ T cells [34], also defective in Nkx2-3 mutant mice [77]. Our preliminary RNAscope observations demonstrate regional differences between the overlap patterns of *Nkx2-3* with *Madcam1* and *Clever1/Stab1* mRNA expression, suggesting compartment-dependent differences in the Nkx2-3 target gene spectrum (Fig. 2).

**Fig. 3:**
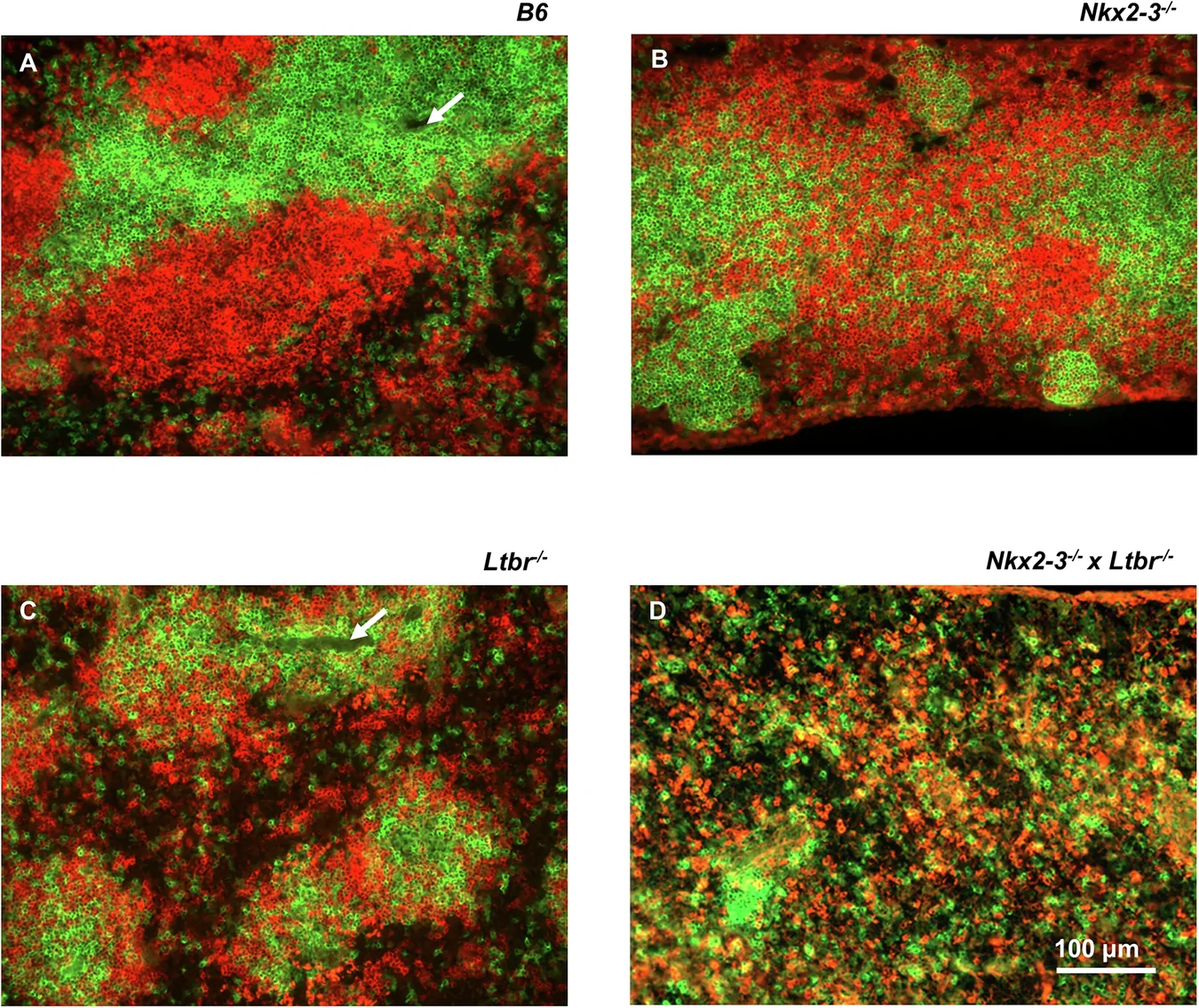
Effect of combined deficiency of Nkx2-3 and LTβR on the lymphocyte distribution in the spleen. Acetone-fixed cryostat sections from young adult mice (genotype indicated at the top) were labeled for T cells (anti-Thy-1.2/CD90, green) and B cells (anti-B220, red). In wild-type C57Bl/6 J mice well-defined T-zona and follicles are distinguishable (**A**), while in *Nkx2-3*^*–/–*^mice non-T/B zones (corresponding to red pulp) are largely absent, with irregular mixing of T and B cells (**B**). In mice lacking LTβR separate red and white pulp (without follicles) are recognizable (**C**), whereas in double mutants T and B cells are randomly scattered without identifiable red pulp/white pulp segregation (**D**). Arrows in (**A, C**) point at central artery. Representative image (*n* = 3).

Another striking difference between the lack of Nkx2-3 and that of LTβR is the ectopic appearance of HEVs expressing PNAd after a switch from early postnatal MAdCAM-1 display, which mediates an L-selectin-dependent homing in Nkx2-3 mutant mice. In addition, the appearance of PNAd^+^ HEVs requires LTβR activity; however, this lymph node-like vascular shift does not indicate complete stromal transition to lymph node-like development, as the spleen, as a separate organ, develops even in (*Nkx2-3*^*–/–*^) x (*Ltbr*^*–/–*^) double mutants, combining the lack of RP vasculature and the absence of MAdCAM-1/PNAd addressin expression [78]. This emergence of rudimentary spleen in double mutants thus reflects the residual developmental potential of LTβR-independent RP fibroblastic scaffolding and Nkx2-3-independent vasculature. Interestingly, the highly disorganized splenic anlage in young adult (*Nkx2-3*^*–/–*^) x (*Ltbr*^*–/–*^) double mutant mice could still harbor lymphocytes, albeit without any identifiable pattern compared to wild-type and parental mutant mice (Fig. 3). As the lack of MAdCAM-1 itself in *Madcam1*^*-/-*^ mice with normal Nkx2-3 levels does not seem to affect the spleen morphology and lymphoid architecture [79], the developmental defects caused by the lack of Nkx2-3 manifest in a MAdCAM-1-independent manner. In contrast, the formation of Peyer’s patches in *Madcam1*^*–/–*^ mice is severely blocked, while the transcriptional absence of MAdCAM-1 in Nkx2-3 deficiency with a similar MAdCAM-1→PNAd HEV addressin switch still permits the formation of Peyer’s patches and other intestinal lymphoid aggregates, with less impairment of maturation [79–81].

To induce MAdCAM-1 in Peyer’s patches HEVs, Nkx2-3 cooperates with COUP-TFII/Nr2f2 to activate *Madcam1* promoter through a composite recognition element, antagonized by a competitive HEY1 docking site [82]. Interestingly, Nkx2-5 also participates in forming a similar complex incorporating COUP-TFII, operational in cardiac development, indicating a conserved regulatory mechanism in an organ-specific Nkx2 expression-dependent manner. Taken together, the lack of Nkx2-3 causes a profound alteration of blood vasculature, including the lack of MZ-zone-associated MAdCAM-1 and RP-associated vascular segments, where the main effect appears to be independent of MAdCAM-1, but manifests through other undefined target genes, and cannot be compensated by preserved Nkx2-5 activity.

While the induction of PNAd in the Nkx2-3-deficient spleens and Peyer’s patches could be regarded as analogous to HEV induction in tertiary lymphoid tissues following lymphocyte-induced transformation [83, 84], we also demonstrated the presence of LYVE-1^+^/Prox1^+^ lymphatic endothelial cell (LEC) meshwork structures, indicating a more fundamental deviation of vascular patterning [77, 85]. Thus, the absence of Nkx2-3 appears to unlock an alternative vascular differentiation by allowing LEC differentiation instead of blood endothelium cell (BEC) and, within the venous BEC, commitment along lymph node-like HEV formation, respectively. Therefore, it appears that the absence of Nkx2-3 pivots the splenic vascular patterning towards a lymph node-like pathway, raising the issue of potential regulatory interaction between Nkx2-3 or its paralogue Nkx2-5 and Prox1 in the organ-specific vascular specification of the spleen, warranting further studies [77]. A similar potential Nkx-Prox1 antagonism was observed in heart formation, where Prox1 haploinsufficiency could rescue the cardiac defects in Nkx2-5 haploinsufficient mice through histone deacetylase-3 (HDAC3) as a corepressor [86]. It also remains to be seen whether the extensive RP impairment also affects the spleen’s capacity to promote sequential hematopoietic reconstitution and myeloid regeneration [87–89].

**Fig. 4:**
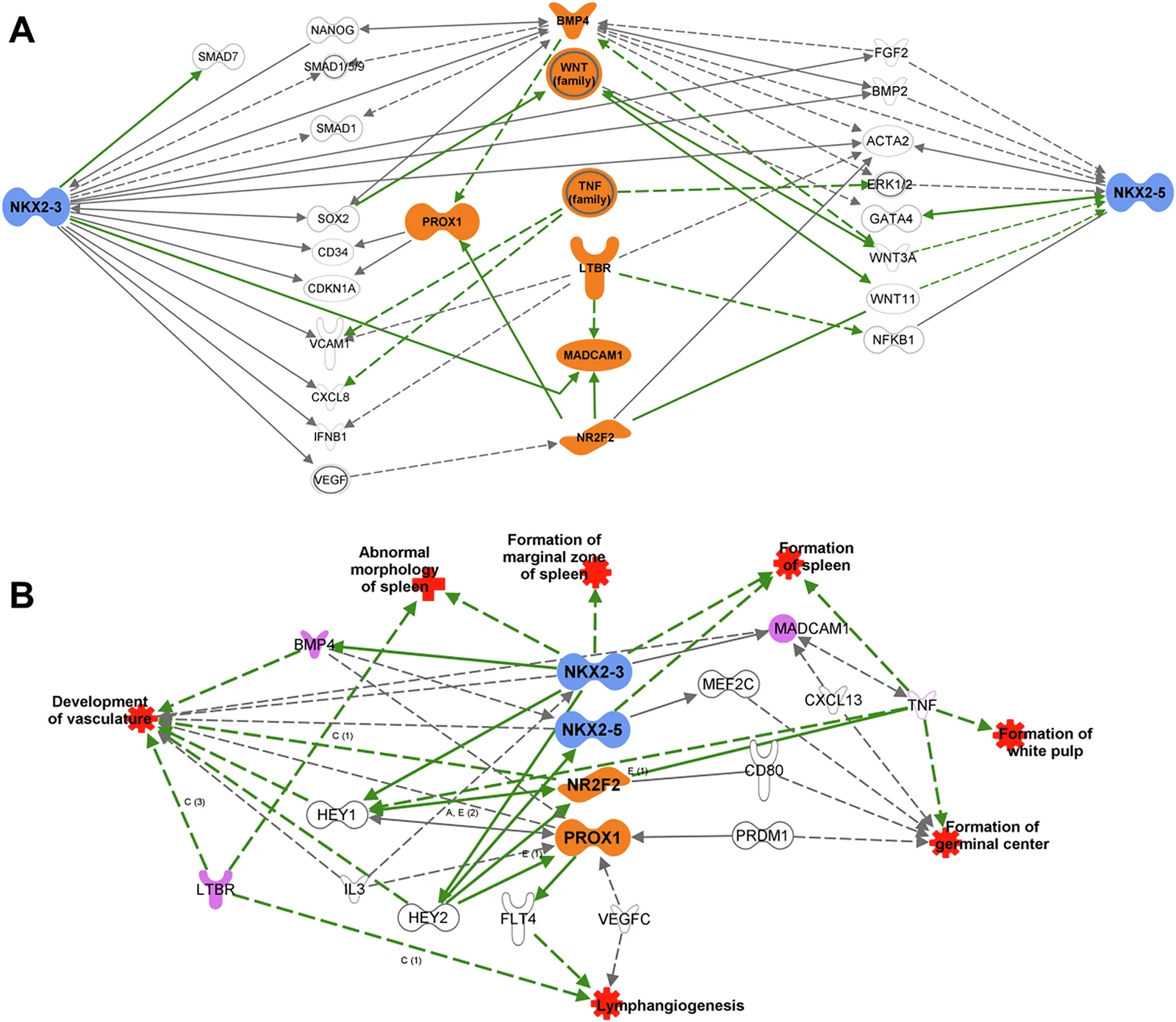
Predictive analysis of molecular pathways involving Nkx2-3 and Nkx2-5 in the development of the spleen. Diagrams were created with the IPA software through the Path Explorer tool (QIAGEN Inc.). **A** Pathways that interact with Nkx2-3 and/or Nkx2-5. **B** Involvement of different molecules in the development and morphogenesis of spleen components and vasculature. The targets of comparison (Nkx2-3 and Nkx2-5) are depicted in blue. Highlighted in orange color are the molecules that interact with the main targets or with intermediate molecules. Continuous lines show direct connections, while dashed lines indicate indirect or incompletely explored links. Green arrows represent relationships exemplified in the text. On **B**, the nodes shown in purple are also present in (**A**). The red nodes designate structural or functional features affected by the other nodes and their interactions.

Predictive modeling with QIAGEN Ingenuity Pathway Analysis (IPA) was used to explore known molecular interactions of Nkx2-3 and Nkx2-5 and possible outcomes on genes and molecules of interest, as well as their predicted roles in disease or altered physiological function through the Path Explorer and Molecule Activity Predictor (MAP) tools. From the several interactions and pathways that have been suggested, the common or overlapping associated molecules between Nkx2-3 and Nkx2-5 are further highlighted (Fig. 4A). Nkx2-3 influences BMP4 expression in both humans and mice, affecting intestine and spleen formation [90], besides the involvement of Nkx2-5 in the relationship between Wnt/β-catenin and BMP4/Activin A signaling pathways in cardiac developmental processes [91]. Furthermore, Wnt family molecules upregulate mouse *Nkx2-5* mRNA levels in different scenarios, namely WNT11 in bone marrow [92] and WNT3A in cardiac muscle [93]. Members of the TNF family also bridge interactions shaped by the Nkx2 paralogs and other intermediate molecules, where decreased activity of human NKX2-3 inhibits expression of VCAM1 and CXCL8 [94] in microvascular endothelial cell lines. On the other hand, inhibition of ERK1/2 reduces the upregulation of *Nkx2-5* mRNA in cardiac differentiation of mouse embryonic stem cells [95], where Nkx2-3 has no apparent effect.

The known relationship between MAdCAM-1 and Nkx2-3 is reinforced when observing that *MAdCAM1* mRNA and protein expressions are regulated by Nkx2-3 in different human and mouse tissues [70, 71, 96]. As mentioned earlier, the expression of MAdCAM1 in the mouse intestine is regulated by COUP-TFII/NR2F2 [82]; however, the interaction of the latter with the Wnt signaling pathway has not been described in humans [97]. Beyond the established connection between the Nkx2 family members and Prox1 and the further interaction between Prox1 and COUP-TFII/NR2F2, it is also proposed that COUP-TFII/NR2F2 is needed for *Prox1* gene activation in mouse lymphatic endothelial cells [98]. In a more general perspective, Nkx2-3 and Nkx2-5 commonly interact with the SMAD and GATA family members of transcription factors, being crucial for the regulation of development and morphogenesis, including cardiac [99] and skeletal muscle [100], as well as in pathologies, such as affecting the intestinal endothelium [96].

In view of the several pathways suggested to interact with the Nkx family, the impact of these transcription factors on the regulation of developmental and morphogenic processes is well determined, and their influence in physiological alterations of structure and/or function can be exemplified (Fig. 4B). Additionally, Nkx2-3 is also correlated with vasculature development through interactions with BMP4, involved in angiogenesis and endothelial cell proliferation [101]. Moreover, Nkx2-3 also affects the expression of HEY1/2 transcriptional regulators, directly associated with mouse vasculogenesis [102]. Interestingly, HEY2 associates with Nkx2-5 [103] and Prox1 [104], indicating the role of these molecules in the development of vasculature as well. Additionally, the proliferation of endothelial cells is affected by COUP-TFII/NR2F2 expression, especially regulating vein commitment [105]. In this scenario, while both HEY1/2 regulate the expression of COUP-TFII/NR2F2 mRNA [104, 106], HEY1 activation and expression may be affected by COUP-TFII/NR2F2 and TNF, respectively [107]. In conclusion, Nkx2-3 and Nkx2-5 engage both shared and distinct partners and targets during the development of spleen vasculature and lymphoid compartments, where they exert partially overlapping but complementary functions, without apparent compensatory potential.

## Stromal aging and splenic restoration—local and systemic immunological impacts and perspectives

Although deciphering general as well as organ-specific pathways of splenic stromal differentiation represents an important progress in understanding the establishment of physiological immune responsiveness, it is equally important to correlate it with various disease situations associated with impaired tissue integrity. The most frequent condition with the simultaneous impairment of immune responsiveness and increased prevalence of autoimmunity is linked to aging, also raising the issue of restoring lymphoid tissue fitness. Importantly, the aging of the immune system also appears to promote systemic aging [108]. In this last section, we briefly overview the characteristics of aging-associated dysregulation of splenic stromal architecture and current trends for its restoration.

Aging as a physiological process has long been recognized as an important condition affecting the organism’s innate and adaptive immunity, including the impairment of the immune system to recognize and eliminate pathogens and establish immunological memory or recall responses following previous exposure. The vast majority of research has addressed the various deviations of hematopoietic cells. On the other hand, research on the effect of “stromal aging” is relatively scarce and has only recently begun to be explored in depth, despite earlier pioneering studies on the importance of FDC aging in the recall responses [109] or prion spreading [110]. These early studies have established that the B cells’ capacity to initiate germinal center reactions is critically influenced by the immune complex retention mechanism and ITIM/ITAM signaling stoichiometry by their FDC environment [111, 112]. Subsequent studies analyzing T-cell responsiveness using similar reciprocal cell transfer experiments indicated that, while the lymphocyte composition is different between the old and young donors, aged recipients fail to sustain homeostatic survival of young naïve donor T cells. However, young hosts can provide a suitable microenvironment for T-cell expansion and cytotoxic immune response [113–115]. On the other hand, in aged mice (older than 18 months), the spleen contains fewer TRCs and produces less T-zone homeostatic chemokines CCL19 and CCL21, leading to a blurred separation of T/B zones and reduced T-zone recruitment of young CD8 + T-cells [116]. In addition, the structural analysis of splenic reticular cell organization in aged mice revealed a discontinuous MZ with a diffuse arrangement of metallophilic macrophages, as well as shrunken and fragmented follicles and FDCs, and a loosened T-zone FRC meshwork. The RP macrophages appeared to relocate to the white pulp and were distributed in a less compact pattern compared to those in young mice [117].

With all those important organ-specific immunological functions that the spleen performs and the complex tissue architecture serving these activities, can the splenic functionality somehow be reinvigorated to correct its gradual deterioration of structure in aging? Following its surgical removal, splenic autotransplantation of small fragments has been used to prevent overwhelming post-splenectomy infection (OPSI) caused by *Streptococcus pneumoniae* or, less frequently, *Haemophilus influenzae*, with a dramatic mortality rate. Using rat transplant models, a consistent tissue rebuilding pattern was noted, where the follicles were regularly regenerated; however, the T-zone and MZ could only regenerate in young hosts, suggesting that the replantation of splenic grafts is more effective in children than in adult patients [118]. Similarly, transplantation of early postnatal mouse splenic capsular stroma could reconstitute the normal splenic architecture, where the presence of a CD31^+^/CD105^+^/CD201^+^/MAdCAM-1^+^ stromal population from the early postnatal spleen was critical for the successful development of heterotopic spleen. Interestingly, these cells lacked Nkx2-5 but expressed LTβR [119].

The significance of LTβR in the development of peripheral lymphoid tissues in humans (in addition to the extensive research in mice) has recently been highlighted by the first identification of patients with biallelic loss-of-function mutations of LTβR causing a hitherto undescribed form of immunodeficiency. Similar to the mouse *Ltbr*^*–/–*^ phenotype, the pediatric patients lacked lymph nodes [120]. Although the impact of the absence of LTβR activity on the spleen structure in these patients has not been reported, the presence of Howell-Jolly bodies in the blood smear of one patient indicates impaired splenic functionality. These findings highlight the importance of considering the impact of stromal differentiation of secondary lymphoid organs in humans with undefined immunodeficiencies and other immunological dysfunctions. More recently, the regenerative potential of human bone marrow mesenchymal stromal cells—an unfractionated population with secretory and immunomodulatory functions but lacking multilineage differentiation and self-renewal capacity typical of mesenchymal stem cells—was tested in a xenogeneic model. The intravenous inoculation of mesenchymal stromal cells could restore splenic immune responsiveness and germinal center (GC) reaction potential through promoting the proliferation and expansion of MAdCAM-1-positive marginal reticular cells (MRCs) by secreting VEGFA [121]. These observations emphasize the important role of MZ stromal architecture and MZ macrophage organization, and the consequence of its structural disintegration in the follicular shuffling of MZ B cells, also impaired in aging [122].

## Concluding remarks

The broadening range of cellular and molecular analyses, together with advanced in vivo mouse models, has deepened our understanding of SLOs as dynamic systems. These systems are maintained by continuous interactions between mobile hematopoietic and sessile stromal components across distinct vascular and extravascular niches. Although SLOs share many features, emerging data reveal molecular cues that drive organ-specific regionalization, with the spleen showing the most complex architecture and compartmentalized developmental and functional diversity of its stromal cells, evidenced by marker expression and mRNA signatures. Further studies are needed to translate the implications of murine tissue-specific stromal specialization to humans beyond structural and phenotypic similarities towards in vivo functions, exploring potential roles for stromal cells in immunopathology and tissue regeneration.
